# No evidence for the utility of negative cues in a visual search-based concealed information test

**DOI:** 10.1186/s40359-026-04594-3

**Published:** 2026-04-22

**Authors:** Tomoya Kawashima, Takashi Kabata

**Affiliations:** 1 Department of Psychology and Information Design, College of Media Information, Kanazawa Institute of Technology, 3-1 Yatsukaho, Hakusan, Ishikawa 924-0838 Japan; 2 Scientific Crime Laboratory, Kanagawa Prefectural Police, Yokohama, Kanagawa Japan

**Keywords:** Concealed information test, Reaction time, Visual search, Cueing distractor, Attention

## Abstract

**Background:**

The Concealed Information Test (CIT) is a widely used method for detecting whether individuals possess knowledge that they are trying to hide. This is typically assessed via physiological or behavioral indicators, such as reaction time. The present study examined whether inefficient visual search behaviorspecifically, slowing when participants are cued to ignore distractor features — can serve as a behavioral marker of concealed information, reflecting attentional capture and suppression processes. This approach was motivated by previous findings showing that cues associated with task-irrelevant items can impair visual search performance.

**Methods:**

Experiment 1 employed a visual search paradigm with distractor cues, based on previous negative cueing studies, to replicate their findings using object-based visual stimuli. Experiments 2 and 3 implemented a visual search–based concealed information test (CIT), in which the memorized and concealed item appeared as a distractor cue in the search display. Reaction times were recorded as the dependent measure.

**Results:**

Experiment 1 confirmed that the use of distractor cues resulted in longer search times, suggesting inefficient visual processing. However, Experiments 2 and 3 revealed no significant differences in search performance between trials with and without concealed cues.

**Conclusions:**

Although visual search under distractor cueing was inefficient, it did not reliably reflect the presence of concealed information. The results suggest that attention to concealed items may be strategically suppressed or redirected, which undermines their detectability in reaction time-based visual searches. These findings emphasize the importance of incorporating alternative markers, such as eye movements or physiological signals, to enhance CIT sensitivity in future research.

## Introduction

The Concealed Information Test (CIT) is a well-established method of detecting whether a person possesses knowledge of specific information (e.g. details of a crime) that they are trying to hide [1, 2]. CIT is traditionally implemented as a psychophysiological test in which suspects are presented with a series of items (one relevant ‘probe’ item among several irrelevant ‘fillers’) and their physiological responses, such as skin conductance, heart rate or pupil dilation, are measured. Recognized crime-relevant items typically elicit different physiological responses to neutral items, enabling the identification of concealed knowledge (e.g. [3]). However, the need for specialized equipment and the possibility of suspects taking countermeasures has prompted researchers to investigate behavioural indicators of concealed knowledge, particularly reaction times.

### Reaction time-based CIT and theoretical background

In reaction time (RT)-based CIT paradigms (often called RT-CIT), participants typically perform a speeded classification task involving a series of stimuli that include relevant (critical concealed items) and irrelevant items. A key finding is that responses to relevant items are significantly slower than responses to irrelevant items [4]. For instance, Seymour et al. [5] demonstrated in a seminal study that reaction times can reliably indicate guilty knowledge: participants took longer to respond when the displayed item was one they recognised and were attempting to conceal, compared to when it was an irrelevant item. This RT-slowing effect in CIT suggests that recognizing a concealed item interferes with the normal response process.

According to the orienting response hypothesis, a relevant item triggers an involuntary orienting response because it is significant or personally relevant to the examinee [6, 7]. The orienting response is a reflexive attentional and physiological reaction to meaningful stimuli. In the CIT context, a guilty person perceives the relevant item (e.g. the murder weapon or their own name) as salient, evoking an enhanced orienting response manifested by attention capture and heightened arousal [8, 9]. This attentional engagement with the relevant item can momentarily delay ongoing task responses, thereby slowing reaction times. In short, the examinee’s attention is pulled towards the familiar relevant item, even if they try to treat it like any other item.

The response conflict hypothesis emphasizes the cognitive conflict and interference that occur when one must conceal knowledge [10, 11]. A person who recognizes the relevant item knows the ‘true’ response (e.g. that the item is familiar), but they must respond as if the item were irrelevant or unfamiliar to hide this knowledge. The contradiction between their automatic recognition and the required deceptive response creates a response conflict, slowing down their reaction time. Even in tasks that do not explicitly request a judgement of familiarity, the relevant item can evoke a predominant response tendency that must be inhibited, resulting in longer RTs. This suggests that while suppressing the truth causes the greatest interference, merely recognizing a significant item can delay responses to some extent (consistent with an orienting effect).

These two perspectives are not mutually exclusive. Contemporary research suggests that both involuntary orienting to meaningful stimuli and active response inhibition cause the RT-CIT effect [10]. In other words, concealed items are harder to ignore for two reasons: (1) their personal significance automatically draws the person’s attention and (2) the person’s attempt to hide recognition creates a cognitive load. These two mechanisms align with the intuitive notion that the information one is trying to hide is mentally ‘sticky’ – it holds attention and is cognitively demanding to suppress.

### Attentional capture by concealed information

If crime-relevant items are indeed automatically attended to, this would be expected to manifest not only in slower responses during a CIT, but also in other measures of attention allocation. There is some evidence that attempting to suppress or ignore a salient, task-irrelevant item can paradoxically make it more prominent in one’s attention. A striking example of this is the ‘search while destroying’ phenomenon described by Kawashima and Matsumoto [12]. In their visual search study, participants were given negative cues, i.e. they were told which features to ignore while searching for a target. The intuitive hope was that knowing what to ignore would help the search by focusing only on relevant items. However, the opposite occurred: when a distractor was cued to be ignored, participants’ attention was automatically drawn to it at the early stage of the search. Participants would fixate on or process the ‘forbidden’ distractor first (as if the mind cannot help but check the item you are trying not to think about) and then had to inhibit it and refocus on the actual target. This process incurred a cost: search performance was poorer with negative cues than with comparable positive cues (where participants were told what the target was). In fact, even in the later stages of the search, the performance remained slower in the negative cue condition, suggesting that the interference from the salient distractors persisted throughout the task. In summary, ignoring a salient item came at a cognitive cost: the very act of trying to ignore it caused attention to dwell on it for longer, which undermined overall task performance.

The analogy with concealed information is clear: a guilty suspect will not react to or think about crime-relevant items presented in a CIT, effectively treating them as distractors to be ignored among innocuous items. The orienting response hypothesis predicts that the concealed item will capture attention involuntarily despite these intentions. Similarly, the search-while-destroying effect suggests that suppression attempts can be ineffective, resulting in delayed disengagement from the item one wishes to disregard. Thus, from an attentional perspective, a relevant item in a CIT could interfere with ongoing mental activity (or another task) simply because the mind ‘notices’ it more, even if no overt physiological response is measured. This line of reasoning has sparked interest in the use of attention-driven effects to detect concealed knowledge in new ways.

### Applying attentional paradigms to CIT

Researchers have started incorporating CIT into traditional attention paradigms to determine whether concealed information can be revealed through attentional performance deficits. One approach involves using spatial attention cues. For instance, Kabata and Kawashima [13] attempted to integrate CIT into a Posner-style spatial cueing task. They exploited the phenomenon of inhibition of return (IOR), whereby a person is typically slower to detect a target at a location that has just been cued (an inhibitory orienting effect). In this study, however, participants were asked to hold a specific number in mind as their concealed piece of information while performing the cueing task. In some trials, the peripheral cue was a concealed number. It was expected that if the cue was a personally significant (relevant) item, attention would be modulated differently compared to a neutral cue. Indeed, in their first experiment, the typical IOR was reduced when the cue represented the participant’s relevant item, suggesting that attention lingered more on that cue than usual. Essentially, the relevant cue attracted attention, causing participants to focus on that location and diminishing the usual inhibitory effect. However, this result proved difficult to replicate consistently; a second experiment did not reveal any significant differences. The authors conclude that this effect is fragile if it exists and that more research is needed to confirm whether spatial attentional cues can consistently reveal concealed knowledge.

Concealed information can influence involuntary eye movements, offering an alternative to reaction time-based approaches in CIT. Fixation durations tend to increase when individuals are instructed to conceal recognition [14, 16]. This suggests that the act of suppression itself recruits additional cognitive control, altering oculomotor behaviour. For instance, Millen et al. [14] revealed that recognition of familiar faces could be reliably detected through various oculomotor markers (e.g., longer fixation durations), regardless of honesty. These findings highlight that robust differences in fixation behaviour can reveal concealed recognition of well-known faces, supporting the notion that face processing is qualitatively distinct when the stimulus is personally meaningful.

Taken together, these studies demonstrate that attentional capture by crime-relevant items is a real phenomenon that can impair performance. Such evidence strengthens the theoretical stance that relevant stimuli draw attention and offers practical avenues for CIT development beyond standard RT or physiological measures.

### The present study and hypothesis

Building on the aforementioned insights, the present study aimed to test whether the ‘search while destroying’ effect of distractor cues could be applied to a CIT scenario. The present study aimed to examine whether crime-relevant items would be more difficult to ignore during a visual search task. We hypothesized that relevant items, even when designated as distractors, would interfere with search performance because they are motivationally salient and potentially resistant to suppression. To investigate this, we designed a task in which participants first learnt a target item and then completed visual searches. Each trial began with a distractor cue — an item that the participants were instructed to ignore while searching for a different target. Importantly, in some conditions, the cue was the relevant item itself (e.g., a secret number or a crime-related image), whereas in others, the cue was an innocuous, unrelated item. We reasoned that if relevant stimuli attract attention, participants would have difficulty suppressing their attention to the cue when it was the relevant item. Importantly, we did not assume a specific stage at which this interference would occur. The influence of relevant cues could arise during the processing of the cue itself, during the subsequent search process, or both. As a result, the impact of relevant cues on search efficiency was treated as an empirical question rather than a strictly directional prediction.

Three experiments were conducted to examine this effect under different conditions. Experiment 1 aimed to replicate the basic ‘search while destroying’ paradigm (as in Kawashima and Matsumoto [12]) using real-world object images instead of abstract shapes and to confirm that negative cueing impairs search in our setup. Experiments 2 and 3 incorporated the concealed information element: participants concealed a specific item and trials were included where that item could appear as the negative cue. In Experiment 3, some trials were included where the relevant item did not appear at all to determine whether any overall slowing could be attributed to maintaining the secret in mind. We hypothesized that, in these CIT conditions, participants would show clear signs of attentional capture by the relevant item, for example slower search times or higher miss rates when the relevant item was present as a distractor.

In preview, the results did not support this hypothesis. In both Experiments 2 and 3, we found no reliable evidence that a concealed distractor cue impaired search performance more than a regular distractor. In other words, the participants did not exhibit the expected additional slowing when the cue to ignore was their hidden item. This was a surprising outcome given the above-mentioned theoretical reasoning and related findings. This suggests that the attentional “stickiness” of the concealed stimulus was not strong enough to produce a measurable search cost under our specific task conditions. These null findings imply that while concealed information may capture attention in some paradigms, its practical utility in a visual search-based CIT appears limited. In this context, the effect might be too subtle or too easily overridden by task strategy. We discuss possible reasons for this discrepancy (e.g., participants adopting strategies to truly ignore the cue or the nature of the stimuli and task timing) and what it means for the broader idea of using attention-based markers in concealed information detection.

## Experiment 1

### Method

#### Participants

We recruited a total of 30 participants (mean age = 21.1 years, *SD* = 1.47) with a monetary compensation of 500 Japanese yen (approximately 3.2 US dollars at that time). The determination of the sample size was executed utilizing G*Power 3.1 [17], referencing the approach outlined by Watson et al. [18], which indicated that the required sample size was 25 participants. Experimental design was preregistered at https://osf.io/pw3gd.

#### Stimuli and apparatus

**Fig. 1:**
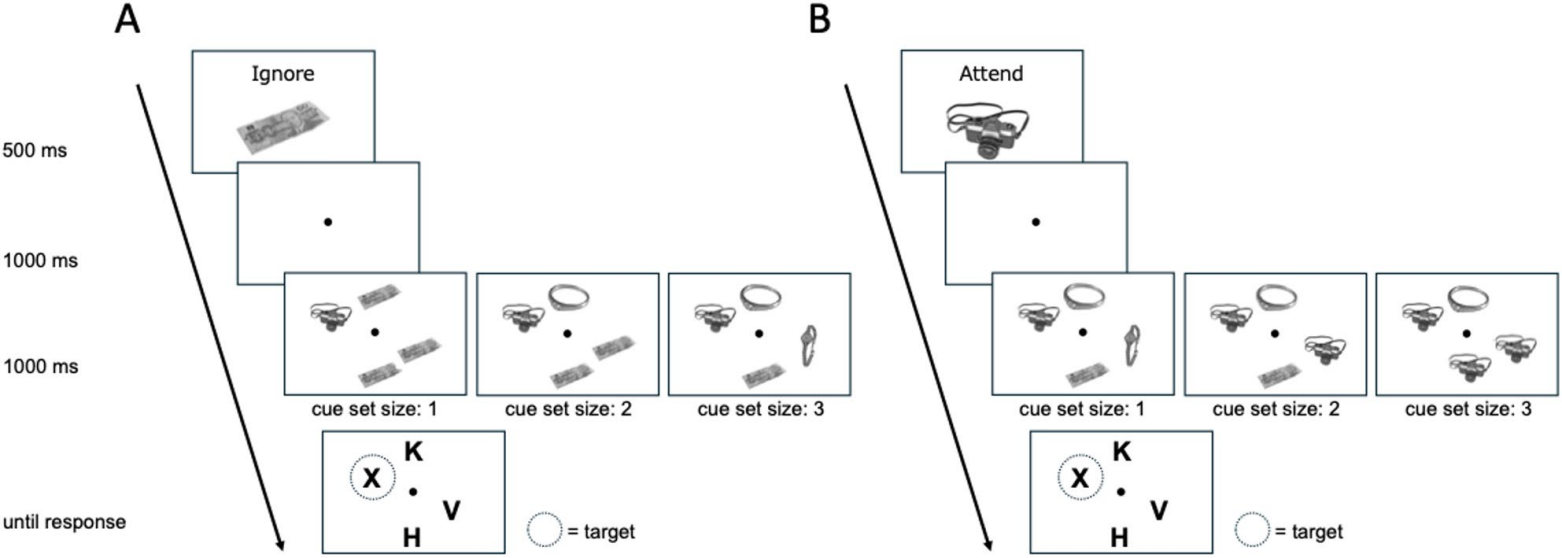
Example trials in the two cue conditions (**A**: negative cue, **B**: positive cue). Participants were instructed to identify whether an “X” or “N” was present as quickly and accurately as possible. Objects presented before the visual search display indicated the locations of the upcoming search letters. Negative cues indicated distractor locations, whereas positive cues indicated potential target locations. In both cue conditions, the cued set size (CSS) was manipulated by varying the number of objects (see text for details)

The visual stimuli were delivered through the Pavlovia.org platform using PsychoPy (v2024.2.2; [19]), following the general procedure described in Kawashima and Matsumoto [12]. Participants completed a short online calibration to estimate screen size and viewing distance [20], from which the visual angles of stimuli were computed. Each trial included the following visual elements: a fixation point at the center of the screen; a centrally presented instruction cue (either a positive cue, such as “Attend [object],” or a negative cue, such as “Ignore [object]”); object cues; and a search display containing four letters arranged on an imaginary circle. Object cues (bill, camera, ring, and watch) were drawn from the BOSS database [21] and presented in grayscale against a white background (Fig. 1).

#### Procedure and design

Each trial began with a 500‑ms cue followed by a fixation display (1000 ms). Participants were instructed that cues were always valid and were to identify a target letter (“X” or “N”) as quickly and accurately as possible. Responses were made via keyboard (“f” key to “X” and “j” key to “N”), and each trial ended with a 500‑ms blank screen before the next one began. The cue type was presented in separate blocks, while the cued set size (CSS) condition varied across trials, with each condition consisting of 24 trials. For both the positive and negative cue conditions, participants completed 72 trials in counterbalanced order, with a short break after every 24 trials. The specific object used as the cue (bill, camera, ring, or watch) varied on a trial-by-trial basis within each block, such that participants were required to both attend to and ignore each object over the course of the experiment. Before the main experiment, participants completed 24 practice trials, where the cue type (positive or negative cue) was randomly varied throughout the practice session.

Cued set size (CSS) was defined as the number of possible target locations remaining after the cue was presented and was manipulated as 1, 2, or 3 by varying the number of cued placeholders. In positive-cue trials (“Attend [color]”), the cue indicated the placeholder locations at which the target could appear, such that the target appeared at one of the cued locations. In contrast, in negative-cue trials (“Ignore [color]”), the cue indicated the placeholder locations to be ignored, and the target appeared at one of the remaining, uncued locations, resulting in an inverse relationship between the number of cued items and CSS. Thus, CSS decreased with more cued items in positive-cue trials, but increased with more cued items in negative-cue trials.

Trials with incorrect target discrimination responses or reaction times that were 3.5 standard deviations away from the mean were excluded from the data analysis, representing 0.97% of the positive cue condition and 1.1% of the negative cue condition. A repeated measures analysis of variance (ANOVA) was conducted for reaction times and accuracy in target discrimination, with 2 levels of the cue type (positive, negative), 3 levels of cued set size (CSS: 1, 2, 3). We adjusted degrees of freedom using the Chi-Muller correction when the assumption of sphericity was violated, and used Shaffer’s modified sequentially rejective Bonferroni procedure for post hoc multiple comparisons.

### Results

#### Reaction times

**Fig. 2:**
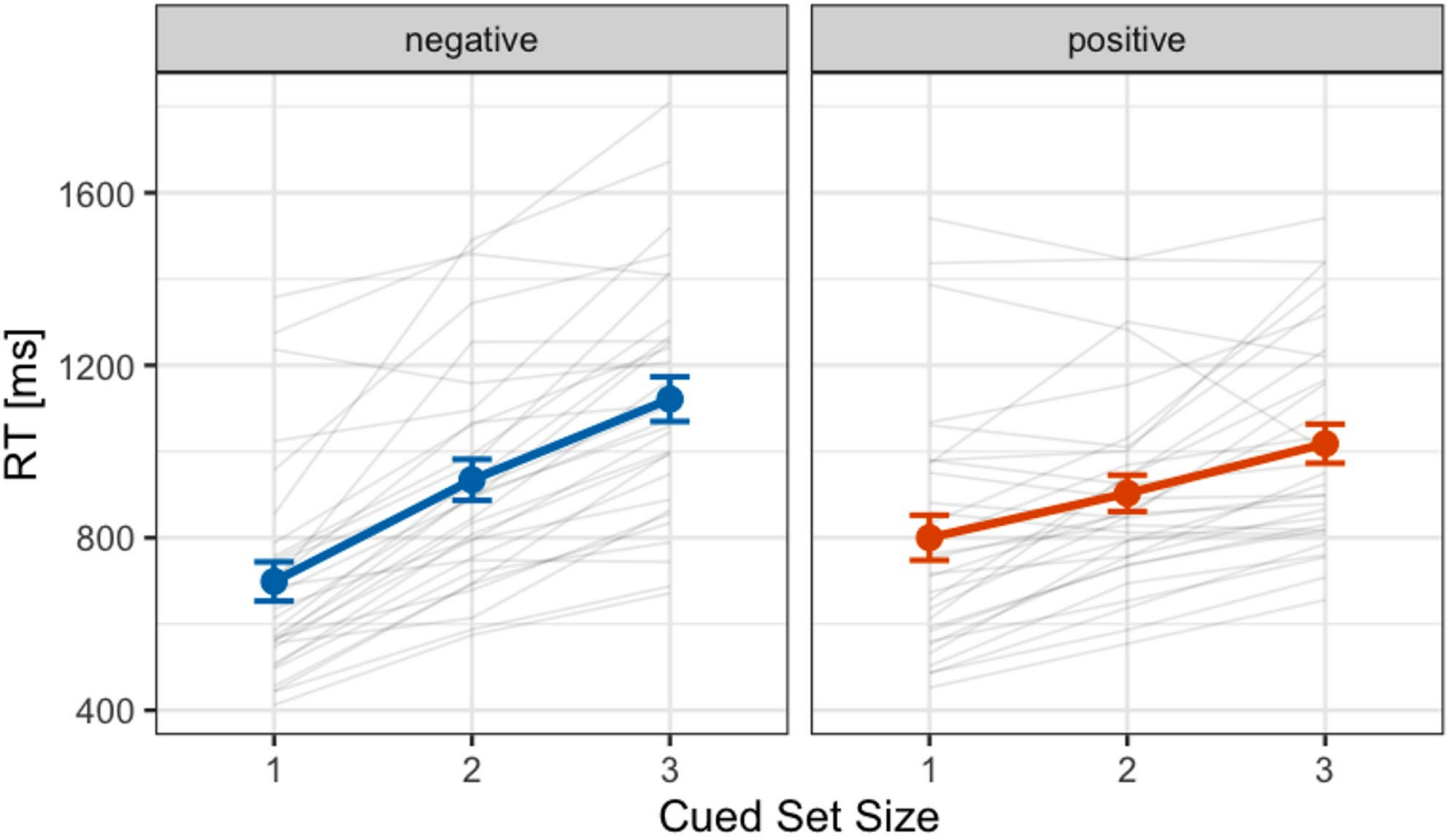
Mean reaction times (ms) for positive and negative cue conditions under the cued set size conditions in Experiment 1. Error bars represent standard errors. Each gray line shows the data of an individual participant included in the final analysis

Figure 2 shows the mean search reaction times as a function of cued set size. A repeated measures ANOVA was conducted with two factors: cue type (positive or negative) and cued set size (1, 2, or 3). The analysis revealed significant main effects for cued set size (*F*(1.44, 41.7) = 91.22, *p* < .001, $\:{\eta\:}_{p}^{2}$ = 0.76) while no significant main effect of cue type (*F*(1, 29) = 0.37, *p* = .546, $\:{\eta\:}_{p}^{2}$ = 0.01). Additionally, a significant two-way interaction was observed (*F*(1.58, 45.81) = 17.96, *p* < .001, $\:{\eta\:}_{p}^{2}$ = 0.38). Analysis of simple main effects showed that at CSS1, the reaction times in negative cue were faster than those in positive cue (*F*(1, 29) = 14.63, *p* < .001, $\:{\eta\:}_{p}^{2}$ = 0.34) and at CSS3, the reaction times in negative cue were slower than those in positive cue (*F*(1, 29) = 11.61, *p* = .002, $\:{\eta\:}_{p}^{2}$ = 0.29). No difference in reaction times in CSS2 between cue types was observed (*F*(1, 29) = 1.77, *p* = .194, $\:{\eta\:}_{p}^{2}$ = 0.06).

We further performed a Bayesian ANOVA to assist in the interpretation of the null findings from the frequentist statistical results using JASP (JASP Team, 2025; jaspstats.org). *BF*_01_ indicates the Bayes factor in favour of H0 (null hypothesis) over H1 (alternative hypothesis). For example, *BF*_01_ = 2 indicates that the null hypothesis is twice as likely to be true as the alternative hypothesis. Consequently, Bayesian ANOVA revealed that the alternative hypothesis (i.e. the presence of a two-way interaction) is 1.157 × 10^5^ times more favoured than the null hypothesis, given our data (*BF*_10_ = 1.157 × 10^5^ in favor of a two-way interaction).

#### Accuracy

The same repeated measures ANOVA was conducted for accuracy data with two factors: cue type (positive or negative) and cued set size (1, 2, or 3). The analysis revealed significant main effects for cued set size (*F*(2, 58) = 5.53, *p* = .006, $\:{\eta\:}_{p}^{2}$ = 0.16) while no significant main effect of cue type (*F*(1, 29) = 2.16, *p* = .152, $\:{\eta\:}_{p}^{2}$ = 0.07) or interaction (*F*(2, 58) = 1.43, *p* = .248, $\:{\eta\:}_{p}^{2}$ = 0.05). Multiple comparisons showed that accuracy at CSS1 was higher than both CSS2 and CSS3 conditions (*t*(29) = 2.55, *p* = .017; *t*(29) = 2.98, *p* = .017) while no significant difference between CSS2 and CSS3 conditions (*t*(29) = 0.81, *p* = .424). Bayesian ANOVA revealed that the null hypothesis (i.e. no two-way interaction) is 2.41 times more favoured than the alternative hypothesis, given our data (*BF*_01_ = 2.41 against a two-way interaction).

**Fig. 3:**
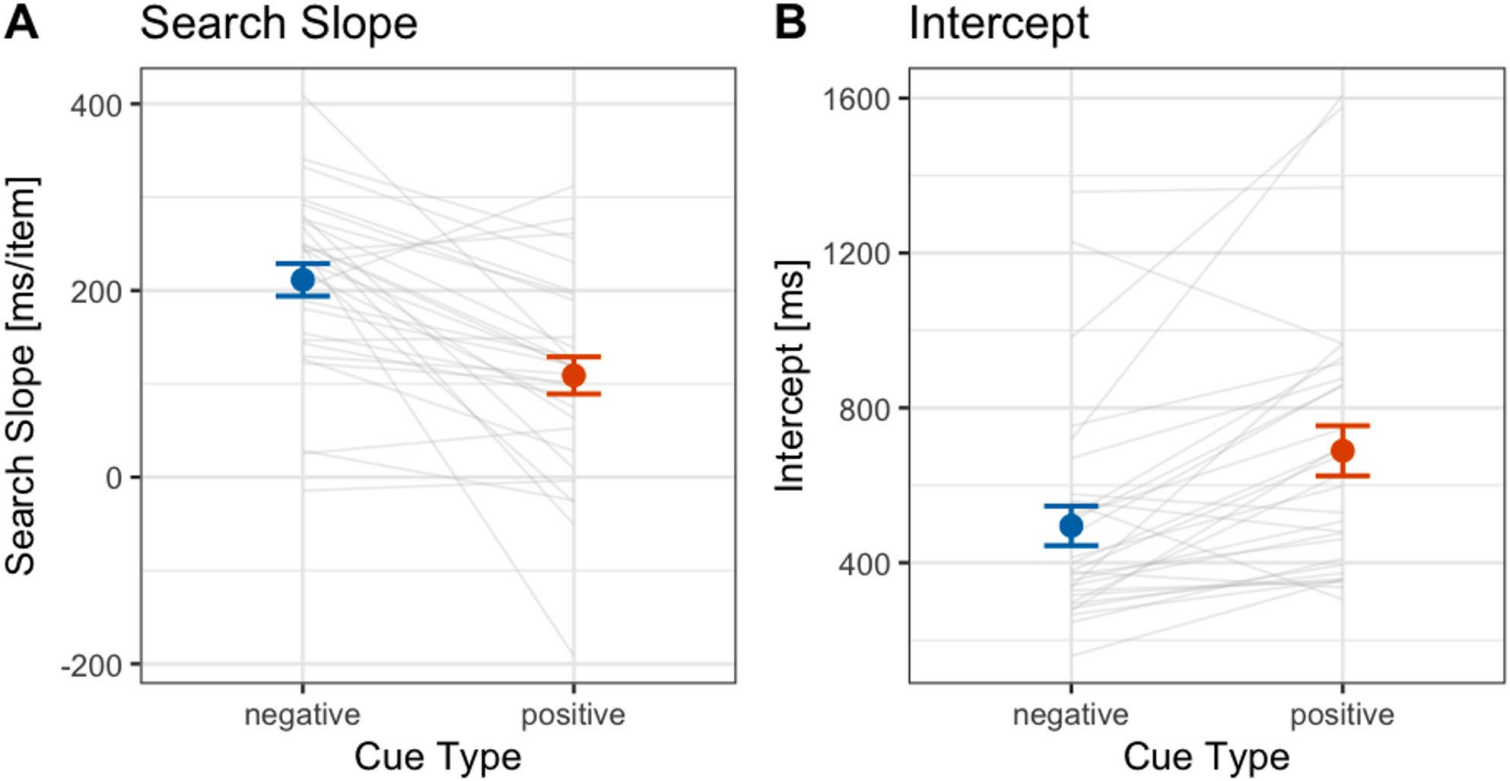
Search slope (**A**) and intercept (**B**) for positive and negative cues in Experiment 1. Error bars represent standard errors. Each gray line shows the data of an individual participant included in the final analysis

#### Search slope and intercept

The search slope was determined by performing a linear regression of the average response times (RTs) for each cued set size (CSS: 1, 2, 3) against the CSS values. The intercept values were obtained from the individual linear regression equations for each participant. Figure 3 shows (A) the mean search slope and (B) the mean intercept, respectively.

Consistent with prior findings and our confirmatory hypothesis for Experiment 1, the search slope in the negative cue condition was significantly steeper than that in the positive cue condition (negative cue: 211.4 ms/item, positive cue: 109.0 ms/item; *t*(29) = 4.75, *p* < .001, Hedges’ *g* = 0.85, 95% CI: 0.43–1.25). For the intercept, unexpectedly, the intercept in the negative cue condition was significantly smaller than that in the positive cue condition (negative cue: 495.3 ms, positive cue: 689.2 ms; *t*(29) = 4.30, *p* < .001, Hedges’ *g* = 0.77, 95% CI: 0.36–1.16).

### Discussion

The results of Experiment 1 replicated the key findings of Kawashima and Matsumoto [12], demonstrating that even when the stimuli are real-world objects rather than simple geometric figures, visual search using negative cues is less efficient than search using positive cues. This suggests that the attentional cost associated with negative cueing is not limited to basic visual features such as color but also extends to more complex object-based representations. Replication with object stimuli underscores the robustness and generalizability of the “search-while-destroying” process proposed by Kawashima and Matsumoto [12].

One notable divergence from the original study was that the intercepts were higher in the positive cue condition than in the negative cue condition. A plausible explanation is that object-based stimuli are generally more difficult to discriminate than colored shapes. Each item in the positive cue condition was different, requiring more effort to locate the target. In contrast, three items shared the same identity in the negative cue condition, which may have facilitated perceptual grouping and allowed participants to quickly exclude them from the search, thereby reducing search time. This grouping-based advantage may have contributed to the lower intercept in the negative cue condition.

Despite this difference in intercepts, the critical finding of Experiment 1 was the replicated increase in search slope under negative cue conditions, indicating reduced search efficiency when participants were required to ignore cued items. This pattern suggests that negative cues induce a state in which attention must be actively suppressed while search is ongoing, consistent with the “search while destroying” framework. Importantly, concealed information in CIT paradigms shares a key functional property with negative cues: although participants are instructed to ignore such information, it remains potentially salient due to its learned or meaningful status. On the basis of this conceptual overlap, Experiment 2 aimed to test whether the attentional inefficiency associated with negative cues could be leveraged for concealed information detection.

Accordingly, Experiment 2 focused exclusively on the negative cue condition from Experiment 1 and introduced a mock-crime manipulation. The concealed object served as the negative cue in the relevant condition, whereas an unrelated object served as the negative cue in the irrelevant condition. Search slopes and intercepts were compared between these two conditions. If concealed information captures attention despite explicit instructions to ignore it, search should be less efficient in the relevant condition than in the irrelevant condition, reflected in steeper search slopes.

## Experiment 2

### Method

#### Participants

We recruited a total of 36 participants with a monetary compensation of 500 Japanese yen (approximately 3.2 US dollars at that time). The determination of the sample size was executed utilizing G*Power 3.1 [17], referencing the approach by Watson et al. [18], which indicated that the required sample size was 33 participants. The effect size *f* for this experiment (= 0.40) was calculated based on the reported effect size of reaction time-based concealed information test (*d* = 1.049) by Suchotzki et al. [22]. The experimental design was preregistered at https://osf.io/cba2r.

#### Stimuli and apparatus

Stimuli and apparatus were identical to those used in Experiment 1, except that only the negative cue was used in Experiment 2.

#### Procedure and design

The procedure was identical to that of Experiment 1, with the following exceptions. Importantly, Experiment 2 included only the negative cue condition; the positive cue condition was not included. The manipulation of cue set size (CSS) and the overall trial structure were identical to those in Experiment 1 and are illustrated in Fig. 1A. For terminological clarity, throughout the manuscript, we use the terms “relevant” and “irrelevant” cues to refer to items corresponding to the concealed (crime-relevant) item and non-corresponding control items, respectively. At the beginning, each participant was assigned one of four objects (bill, camera, ring, or watch) as the stolen item (i.e., crime-relevant item). The assignment of the four objects was counterbalanced across participants. Participants were instructed to read a scenario in which they played the role of a thief who had stolen an item from a box. They were required to memorize the stolen item and then perform the experimental task while concealing their knowledge of the item. In addition, they were informed that a recognition phase would take place at the end of the experiment, during which they would be asked to recall the stolen item. Participants were explicitly instructed to maintain memory for the stolen item throughout the experiment and to remain committed to concealing it while performing the task. In the recognition phase, all four items were displayed on the screen and participants were asked to select the item they had been concealing.

The main experiment consisted of six blocks of 24 trials each, for a total of 144 trials. Of these, 36 were relevant trials (1 item × 3 CSS × 12 repetitions), while 96 were irrelevant trials (3 items × 3 CSS × 12 repetitions). Trials were presented in a randomized order, with a short break after every 24 trials. Prior to the main experiment, participants completed 24 practice trials in which the four objects were presented randomly as cues.

Trials with incorrect target discrimination responses or reaction times that were 3.5 standard deviations away from the mean were excluded from the data analysis, representing 0.3% of the relevant trials and 0.7% of the irrelevant trials. A repeated measures analysis of variance (ANOVA) was conducted for reaction times and accuracy in target discrimination, with 2 levels of the object type (relevant, irrelevant), 3 levels of cued set size (CSS: 1, 2, 3). We adjusted degrees of freedom using the Chi-Muller correction when the assumption of sphericity was violated, and used Shaffer’s modified sequentially rejective Bonferroni procedure for post hoc multiple comparisons.

### Results

Twelve participants were excluded from the analysis due to recognition errors, and one additional participant was excluded due to extremely slow reaction times (over 4,000 ms). As a result, the final analysis included 23 participants (mean age = 19.1 years, *SD* = 1.95).

#### Reaction times

**Fig. 4:**
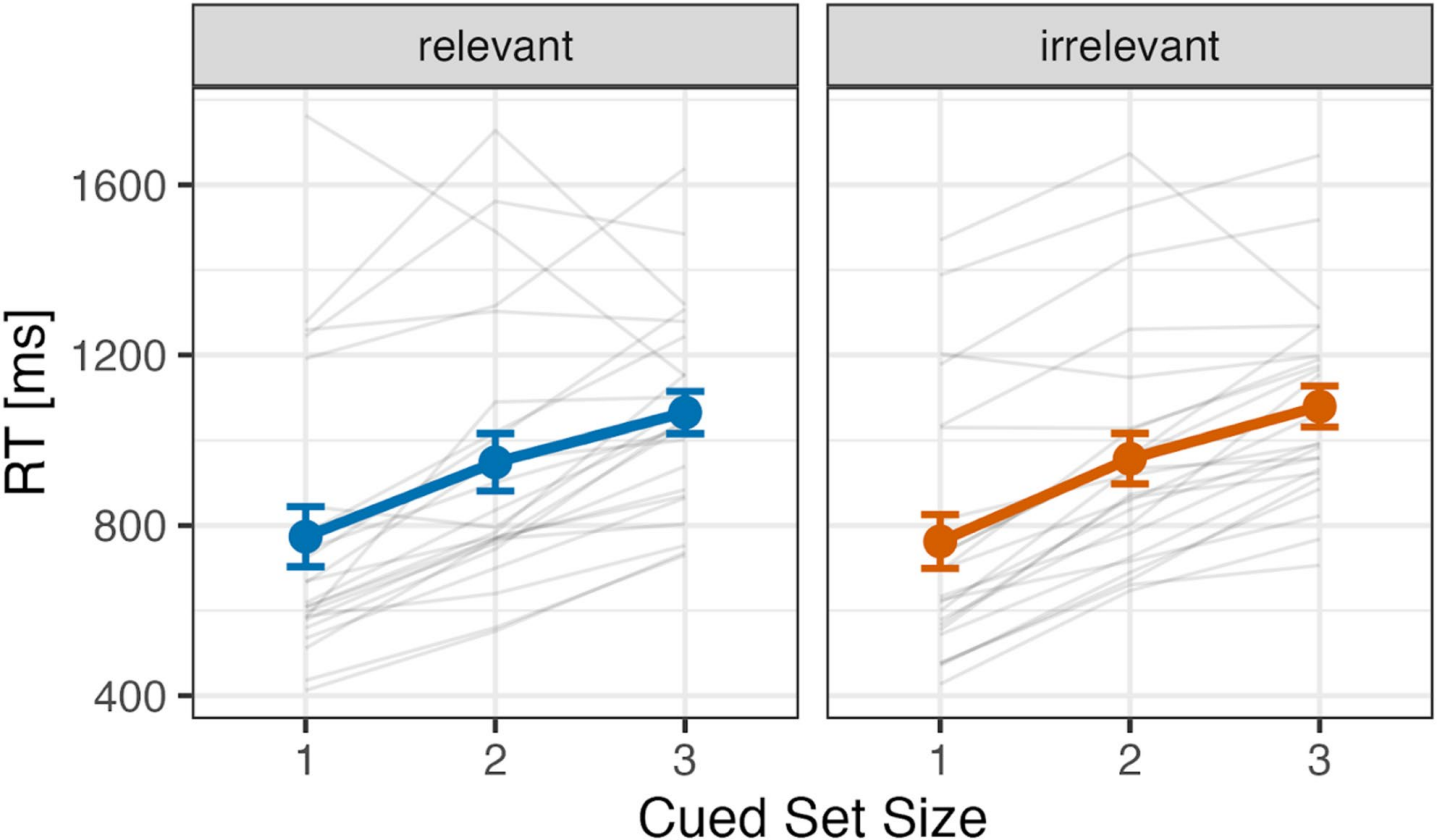
Mean reaction times (ms) for relevant and irrelevant cue conditions under the cued set size conditions in Experiment 2. Error bars represent standard errors. Each gray line shows the data of an individual participant included in the final analysis

Figure 4 shows the mean search reaction times as a function of cued set size. A repeated measures ANOVA was conducted with two factors: cue type (relevant or irrelevant) and cued set size (1, 2, or 3). The analysis revealed significant main effects for cued set size (*F*(1.28, 28.12) = 47.54, *p* < .001, $\:{\eta\:}_{p}^{2}$ = 0.68). Multiple comparisons showed that reaction times in the CSS1 condition were significantly faster than those in CSS2 and CSS3 conditions (*t*(22) = 7.40, *p* < .001; *t*(22) = 9.27, *p* < .001), and reaction times in CSS2 were faster than those in CSS3 condition (*t*(22) = 4.02, *p* < .001). No significant main effect of cue type (*F*(1, 22) = 0.04, *p* = .840, $\:{\eta\:}_{p}^{2}$ = 0.02) nor two-way interaction (*F*(2, 44) = 0.28, *p* = .760, $\:{\eta\:}_{p}^{2}$ = 0.01) was observed. Furthermore, Bayesian ANOVA revealed that the null hypothesis (i.e. no two-way interaction) is 6.37 times more favoured than the alternative hypothesis, given our data (*BF*_01_ = 6.37 against a two-way interaction).

#### Accuracy

The same repeated measures ANOVA was conducted for accuracy data with two factors: cue type (relevant or irrelevant) and cued set size (1, 2, or 3). The analysis revealed no main effects for cued set size (*F*(1.68, 36.96) = 2.76, *p* = .085, $\:{\eta\:}_{p}^{2}$ = 0.11), no significant main effect of cue type (*F*(1, 22) = 0.39, *p* = .537, $\:{\eta\:}_{p}^{2}$ = 0.02), and no significant interaction (*F*(1.7, 37.31) = 1.10, *p* = .333, $\:{\eta\:}_{p}^{2}$ = 0.05). Furthermore, Bayesian ANOVA revealed that the null hypothesis (i.e. no two-way interaction) is 2.97 times more favoured than the alternative hypothesis, given our data (*BF*_01_ = 2.97 against a two-way interaction).

#### Search slope and intercept

**Fig. 5:**
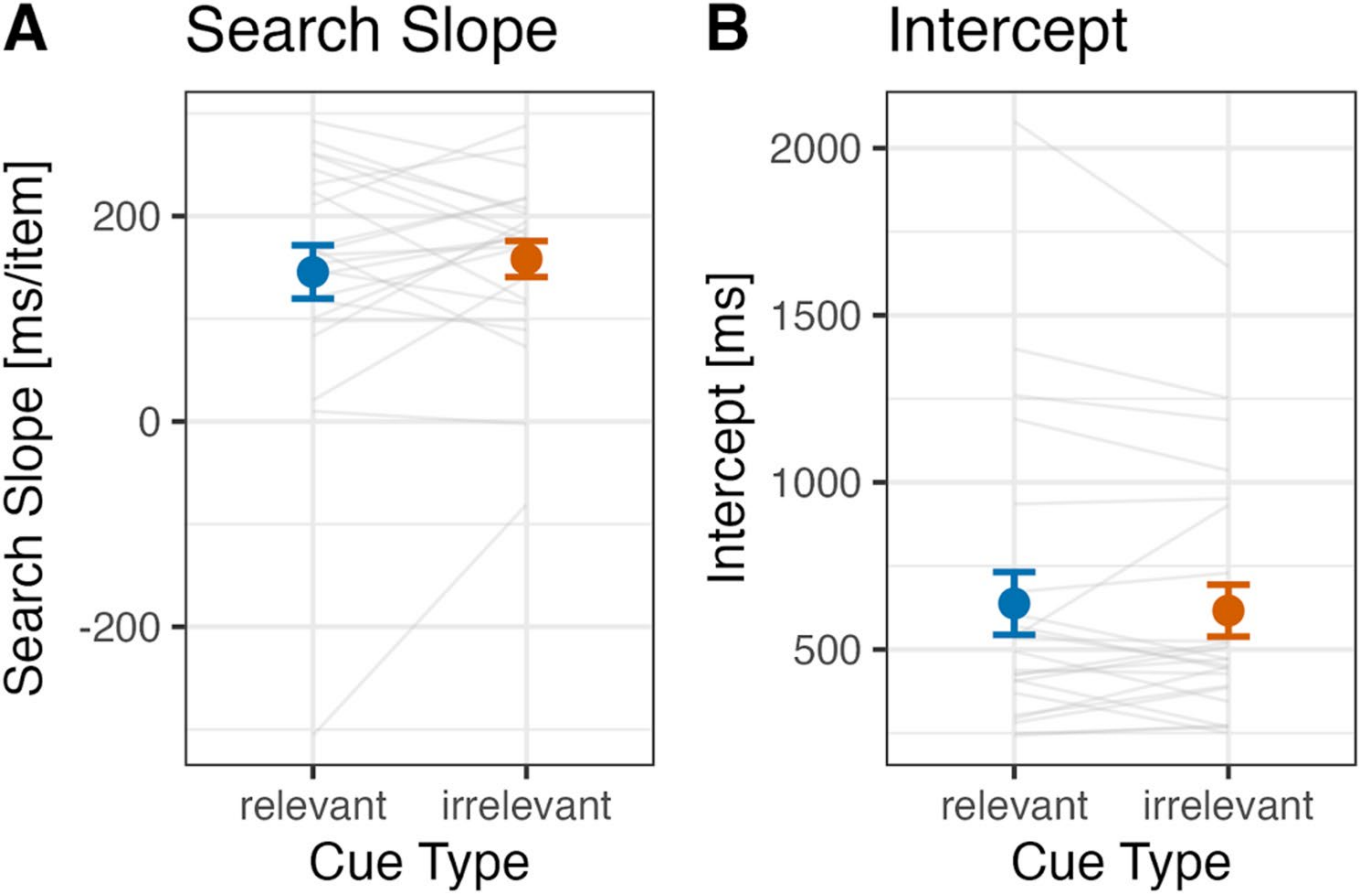
Search slope (**A**) and intercept (**B**) for relevant and irrelevant cues in Experiment 2. Error bars represent standard errors. Each gray line shows the data of an individual participant included in the final analysis

The search slope was determined by performing a linear regression of the average response times (RTs) for each cued set size (CSS: 1, 2, 3) against the CSS values. The intercept values were obtained from the individual linear regression equations for each participant. Figure 5 shows (A) the mean search slope and (B) the mean intercept, respectively.

For the search slope, no significant difference between cue types was observed (relevant cue: 145.6 ms/item, irrelevant cue: 158.2 ms/item; *t*(22) = 0.76, *p* = .458, Hedges’ *g* = 0.15, 95% CI: -0.25–0.55). For the intercept, no significant difference between cue types was observed (relevant cue: 637.8 ms, irrelevant cue: 616.3 ms; *t*(22) = 0.65, *p* = .522, Hedges’ *g* = 0.13, 95% CI: -0.27–0.53).

### Discussion

In Experiment 2, we investigated whether the CIT could be applied to visual search using negative cues. The results showed no significant differences in either the search slope or intercept between conditions in which the relevant item was presented as a cue and those in which it was not. This suggests that detecting concealed information in visual search based solely on reaction times may not be sufficient. However, despite the lack of significant differences, 12 participants made errors in the recognition task, indicating that maintaining the concealed information in this experimental context imposed a high cognitive load. Therefore, in Experiment 3, we aimed to facilitate encoding by presenting the relevant items as object images and verbal information. Furthermore, by including visual search blocks in which the relevant stimuli never appeared, we sought to establish a baseline to examine whether specific responses occurred only when cues matched the concealed items.

## Experiment 3

### Method

#### Participants

A total of 36 participants were recruited and received course credit for their participation. The determination of the sample size was the same as in Experiment 2, which indicated that 33 participants were required. The experimental design was preregistered at https://osf.io/9a3wq.

#### Stimuli and apparatus

Stimuli and apparatus were identical to those used in Experiment 2. In addition, four new images (bag, binoculars, headphones, and laptop) were added in Experiment 3, retrieved from the BOSS database [21].

#### Procedure and design

Experiment 3 consisted of two tasks: the conceal task and the non-conceal task. The order of these tasks was counterbalanced across participants. While the task procedure itself was identical across both conditions, the set of presented images differed, as described below.

At the beginning of the experiment, participants were instructed to read a scenario in which they had stolen one item (either a bill, camera, ring, or watch; counterbalanced across participants), memorize it, and perform the task while concealing that information. Unlike in Experiment 2, the stolen item was presented not only as an image but also as a written word. Participants were informed that a recognition test would be conducted at the end of the experiment. Accordingly, they were instructed to remember the stolen item throughout the session and to try to hide this knowledge while performing the task.

In the conceal task, one of the items mentioned in the scenario was included among the stimuli, replicating the procedure of Experiment 2. The task consisted of 120 trials (30 trials for the related item condition and 90 trials for the unrelated item condition), preceded by 12 practice trials. Participants were allowed to take a self-paced break every 40 trials.

In the non-conceal task, none of the items the participants had been instructed to conceal were presented. In this task, the labels “relevant” and “irrelevant” were assigned by the experimenter on a participant-by-participant basis and did not reflect crime-related or concealed information; these assignments were counterbalanced across participants. Crucially, participants were never informed of any “relevant” or “irrelevant” distinction in the non-conceal task; this classification was applied exclusively at the analysis stage for comparison with the conceal task. Apart from the stimuli, all other aspects of the task—including the number of trials—were identical to the conceal task. In this experiment, two sets were used: bill, camera, ring, and watch, and bag, binoculars, headphones, and laptop. The assignment of these stimulus sets to the conceal and non-conceal tasks was counterbalanced across participants.

After completing both tasks, a recognition test was administered. In this test, participants were asked to identify the item they had concealed by selecting it from a screen displaying all eight images used across the experiment (bill, camera, ring, watch, bag, binoculars, headphones, and laptop).

Trials with incorrect target discrimination responses or reaction times that were 3.5 standard deviations away from the mean were excluded from the data analysis, representing 0.2% of the relevant trials and 0.7% of the irrelevant trials. A repeated measures analysis of variance (ANOVA) was conducted for reaction times and accuracy in target discrimination, with 2 levels of the object type (relevant, irrelevant), 3 levels of cued set size (CSS: 1, 2, 3) for conceal task and non-conceal task, respectively. We adjusted degrees of freedom using the Chi-Muller correction when the assumption of sphericity was violated, and used Shaffer’s modified sequentially rejective Bonferroni procedure for post hoc multiple comparisons.

### Results and discussion

Seven participants were excluded from the analysis due to recognition errors, and one additional participant was excluded for having a low accuracy rate (below 50%). As a result, the final analysis included 28 participants (mean age = 19.0 years, *SD* = 0.60).

#### Reaction times

##### Conceal task

**Fig. 6:**
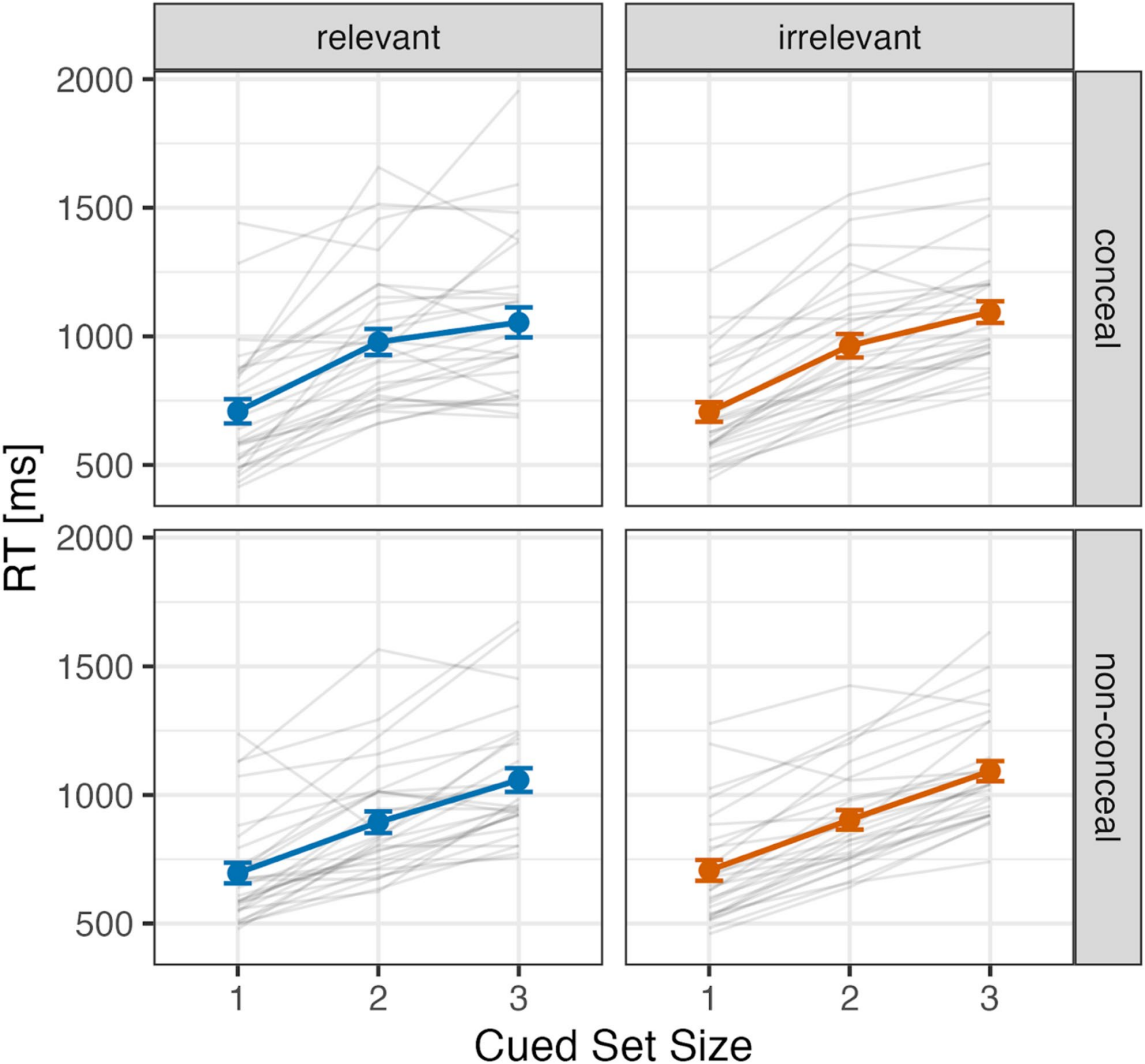
Mean reaction times (ms) for relevant and irrelevant cue conditions across cued set sizes in the conceal and non-conceal tasks (Experiment 3). Error bars represent standard errors. Each gray line shows the data of an individual participant included in the final analysis

Figure 6 (top panel) shows the mean search reaction times in the conceal task as a function of cued set size. A repeated measures ANOVA was conducted with two factors: cue type (relevant or irrelevant) and cued set size (1, 2, or 3). The analysis revealed significant main effects for cued set size (*F*(2, 54) = 117.83, *p* < .001, $\:{\eta\:}_{p}^{2}$ = 0.81). Multiple comparisons showed that reaction times in the CSS1 condition were significantly faster than those in CSS2 and CSS3 conditions (*t*(27) = 11.56, *p* < .001; *t*(27) = 13.67, *p* < .001), and reaction times in CSS2 were faster than those in CSS3 condition (*t*(27) = 4.29, *p* < .001). No significant main effect of cue type (*F*(1, 27) = 0.19, *p* = .663, $\:{\eta\:}_{p}^{2}$ = 0.01) nor two-way interaction (*F*(2, 54) = 1.41, *p* = .253, $\:{\eta\:}_{p}^{2}$ = 0.05) was observed. Bayesian ANOVA revealed that the null hypothesis (i.e. no two-way interaction) is 3.09 times more favoured than the alternative hypothesis, given our data (*BF*_01_ = 3.09 against a two-way interaction). Thus, again, we found no reliable evidence that visual search performance reflects crime-related memory.

##### Non-conceal task

Figure 6 (bottom panel) shows the mean search reaction times in the non-conceal task as a function of cued set size. A repeated measures ANOVA was conducted with two factors: cue type (relevant or irrelevant) and cued set size (1, 2, or 3). The analysis revealed significant main effects for cued set size (*F*(1.68, 45.29) = 119.43, *p* < .001, $\:{\eta\:}_{p}^{2}$ = 0.82). Multiple comparisons showed that reaction times in the CSS1 condition were significantly faster than those in CSS2 and CSS3 conditions (*t*(27) = 8.86, *p* < .001; *t*(27) = 12.71, *p* < .001), and reaction times in CSS2 were faster than those in CSS3 condition (*t*(27) = 8.88, *p* < .001). No significant main effect of cue type (*F*(1, 27) = 2.42, *p* = .131, $\:{\eta\:}_{p}^{2}$ = 0.08) nor two-way interaction (*F*(2, 54) = 0.59, *p* = .561, $\:{\eta\:}_{p}^{2}$ = 0.02) was observed. Bayesian ANOVA revealed that the null hypothesis (i.e. no two-way interaction) is 5.81 times more favoured than the alternative hypothesis, given our data (*BF*_01_ = 5.81 against a two-way interaction).

#### Accuracy

##### Conceal task

The same repeated measures ANOVA was conducted for accuracy data with two factors: cue type (relevant or irrelevant) and cued set size (1, 2, or 3). The analysis revealed no main effects for cued set size (*F*(2, 54) = 1.97, *p* = .149, $\:{\eta\:}_{p}^{2}$ = 0.07), no significant main effect of cue type (*F*(1, 27) = 1.38, *p* = .251, $\:{\eta\:}_{p}^{2}$ = 0.05), and no significant interaction (*F*(2, 54) = 0.29, *p* = .749, $\:{\eta\:}_{p}^{2}$ = 0.01). Bayesian ANOVA revealed that the null hypothesis (i.e. no two-way interaction) is 7.38 times more favoured than the alternative hypothesis, given our data (*BF*_01_ = 7.38 against a two-way interaction).

##### Non-conceal task

The same repeated measures ANOVA was conducted for accuracy data with two factors: cue type (relevant or irrelevant) and cued set size (1, 2, or 3). The analysis revealed significant main effects for cued set size (*F*(2, 54) = 9.32, *p* < .001, $\:{\eta\:}_{p}^{2}$ = 0.26) while no significant main effect of cue type (*F*(1, 27) = 0.001, *p* = .970, $\:{\eta\:}_{p}^{2}$ = 0.0001) or interaction (*F*(2, 54) = 0.03, *p* = .969, $\:{\eta\:}_{p}^{2}$ = 0.001). Multiple comparisons showed that accuracy at CSS1 was higher than both CSS2 and CSS3 conditions (*t*(27) = 2.49, *p* = .019; *t*(27) = 4.14, *p* < .001) while no significant difference between CSS2 and CSS3 conditions (*t*(27) = 1.94, *p* = .063). Bayesian ANOVA revealed that the null hypothesis (i.e. no two-way interaction) is 10.0 times more favoured than the alternative hypothesis, given our data (*BF*_01_ = 10.0 against a two-way interaction).

#### Search slope and intercept

**Fig. 7:**
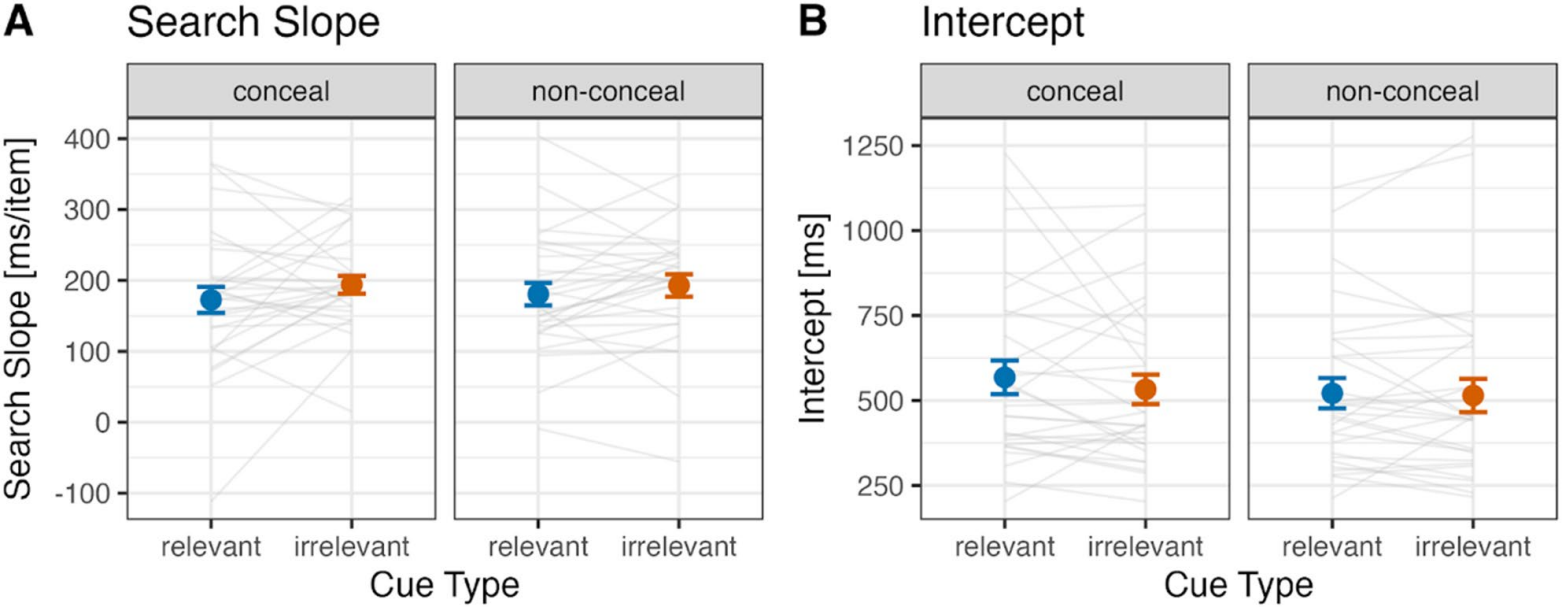
Search slope (**A**) and intercept (**B**) for relevant and irrelevant cues under conceal and non-conceal tasks in Experiment 3. Error bars represent standard errors. Each gray line shows the data of an individual participant included in the final analysis

The search slope was determined by performing a linear regression of the average response times (RTs) for each cued set size (CSS: 1, 2, 3) against the CSS values. The intercept values were obtained from the individual linear regression equations for each participant. Figure 7 shows (A) the mean search slope and (B) the mean intercept, respectively.

##### Conceal task

For the search slope, no significant difference between cue types was observed (relevant cue: 172.7 ms/item, irrelevant cue: 194.1 ms/item; *t*(27) = 1.30, *p* = .204, Hedges’ *g* = 0.24, 95% CI: -0.13–0.60). For the intercept, no significant difference between cue types was observed (relevant cue: 568.3 ms, irrelevant cue: 533.0 ms; *t*(27) = 1.00, *p* = .326, Hedges’ *g* = 0.18, 95% CI: -0.18–0.55). Here, again, we observed no significant effect of crime-related memory on visual search performance.

##### Non-conceal task

For the search slope, no significant difference between cue types was observed (relevant cue: 180.7 ms/item, irrelevant cue: 192.9 ms/item; *t*(27) = 1.02, *p* = .317, Hedges’ *g* = 0.19, 95% CI: -0.18–0.55). For the intercept, no significant difference between cue types was observed (relevant cue: 235.2 ms, irrelevant cue: 260.2 ms; *t*(27) = 0.27, *p* = .787, Hedges’ *g* = 0.05, 95% CI: -0.31–0.41).

## General discussion

This study investigated whether the inefficiency of attentional suppression observed in visual search paradigms could also be applied to the detection of concealed information. In Experiment 1, we replicated the finding of Kawashima and Matsumoto [12] that participants took longer to search when trying to suppress attention to previously cued, task-irrelevant stimuli. Importantly, we extended these findings by using object stimuli rather than color features. Our results confirmed that the cueing inefficiency effect is not limited to color dimensions, thus supporting the robustness and generalisability of the original phenomenon.

Experiments 2 and 3 explored whether this attentional inefficiency could indicate concealed information in a visual search task by embedding crime-relevant items as negative cues. However, we found no reliable evidence that the reaction times to crime-relevant items differed significantly from those to irrelevant items when participants were instructed to conceal their knowledge. Therefore, while Experiment 1 validated the underlying attentional phenomenon, our attempt to extend this mechanism to a concealed information test context was unsuccessful.

One possible explanation for the null effects observed in Experiments 2 and 3 concerns the interaction between attentional capture and top-down inhibitory control under conditions of explicit task irrelevance. In the present task, participants were explicitly instructed to ignore the cue stimulus, and the cue never required a response. This differs fundamentally from standard RT-based CIT paradigms, in which probe stimuli are task-relevant and often require a distinct response, thereby encouraging deeper semantic processing. Under such “ignore” instructions, attentional control may operate at an early stage of processing, limiting the depth to which the cue is analyzed. It is possible that the cue was processed only at a feature- or object-based level, without engaging the semantic processing necessary to identify it as the concealed, crime-relevant item. In this case, even if concealed items possess intrinsic salience or familiarity, their potential to capture attention at a semantic level may have been attenuated by top-down inhibitory mechanisms triggered by task instructions. This interpretation is consistent with previous findings suggesting that concealed relevant information can be actively suppressed. For example, Rosenzweig and Bonneh [23] showed that concealed information modulated oculomotor behavior in a manner indicative of inhibition, and Millen et al. [14, 15, 24] reported reduced fixations on familiar faces when participants attempted to conceal recognition. Taken together, these findings suggest that in the present paradigm, attentional attraction by concealed cues and instruction-driven inhibitory control may have co-occurred, with inhibition operating sufficiently early to prevent reliable behavioral effects on reaction time. Thus, the absence of RT differences in Experiments 2 and 3 does not necessarily imply the absence of attentional capture by concealed items per se, but may instead reflect the strong influence of task relevance and processing depth in determining whether such capture manifests at the behavioral level.

Another possible reason why the effect of the related item was not detected is that the related item was also presented in the search display even in the conditions in which it did not serve as a spatial cue. Specifically, some trials included conditions where the target appeared at the location of the related item, although the related item itself was not presented as a cue. To examine this possibility, we compared the reaction times between trials in which the target appeared at the location of the related item (target_at_relevant condition) and those in which it appeared elsewhere (target_at_irrelevant condition), within the irrelevant cue condition. We performed ANOVA (relevance × CSS) on RT data in Experiment 2 and found no significant main effect of relevance (*F*(1, 22) = 0.29, *p* = .595, $\:{\eta\:}_{p}^{2}$ = 0.01) nor interaction (*F*(2, 44) = 0.58, *p* = .562, $\:{\eta\:}_{p}^{2}$ = 0.03), suggesting that the presence of the related item in the search display did not affect search efficiency.

A critical distinction between our paradigm and typical CIT paradigms lies in the task relevance of the concealed item. In standard CIT tasks, crime-relevant items are meaningful and directly tied to the task (e.g., “Is this your stolen object?”), which naturally elicits attentional capture. However, in our task, the relevant item served as a negative cue—an element that participants were explicitly told to disregard. Participants may have adopted a top-down strategy to intentionally suppress any processing of the cue, including crime-relevant cues. This “strategic ignorability” may have further reduced any observable effects of concealed information, making it less likely to manifest in reaction time.

Another important limitation concerns the strength and reliability of memory encoding for the probe crime-relevant items. In Experiment 2, 12 of 30 participants failed to recognize the concealed relevant item in a subsequent recognition task, indicating that the mock-crime manipulation did not induce a sufficiently strong or stable memory trace for a substantial portion of the sample. This raises the possibility that the absence of reaction-time effects reflects insufficient memory representation of the probe item itself, rather than a genuine absence of attentional capture by concealed information. In Experiment 3, we attempted to strengthen encoding by adding a text label to the cue; however, recognition failures still occurred in 7 participants. These high exclusion rates must therefore be regarded as a major limitation of the present study. In standard CIT paradigms, probe items are typically highly salient, well-learned, and often personally or emotionally meaningful. In contrast, the present mock-crime scenario involved minimal engagement and limited personal relevance, which may have resulted in comparatively weak memory representations. Accordingly, the null findings in Experiments 2 and 3 should be interpreted with caution. They do not allow a definitive conclusion as to whether concealed items fail to capture attention, or whether the experimental manipulation was insufficient to elicit robust concealed knowledge in all participants. These considerations highlight the importance of ensuring strong and verifiable memory encoding when investigating attentional effects of concealed information, particularly when drawing parallels to real-world CIT applications.

An additional limitation concerns statistical power after participant exclusions. Although the planned sample sizes were determined a priori based on effect sizes reported in previous studies, the final sample sizes in Experiments 2 and 3 (*n* = 23 and *n* = 28, respectively) fell below these targets (*n* = 33) due to high exclusion rates. As a result, the present experiments may have been underpowered to detect smaller-than-expected effects, particularly interaction effects. This limitation is especially relevant given that the hypothesized influence of concealed items on search performance may be subtle rather than large. Therefore, the absence of significant effects in the frequentist analyses should be interpreted with caution, as small effects cannot be definitively ruled out with the current sample sizes. To address this issue, we complemented the repeated-measures ANOVAs with Bayesian analyses, which allowed us to quantify the evidence in favor of the null hypothesis. These analyses provided moderate to strong evidence against the presence of the critical interaction effects, suggesting that, even if such effects exist, they are likely to be smaller than the effects originally anticipated. Nevertheless, future studies employing larger samples and more robust memory encoding procedures will be necessary to more sensitively test for small effects in this paradigm.

In the current task, it is possible that, in addition to attentional capture by relevant stimuli, the structural properties of the search display itself also affected search efficiency. As a result, these two effects may have counteracted each other, leading to an overall null effect. Specifically, in the negative-cue condition with CSS = 1, all but one item in the search display were identical, resulting in a relatively homogeneous background. Under such conditions, a single deviant item may have produced a pop-out-like effect, thereby facilitating visual search. Indeed, in Experiment 1 of the present study, the negative-cue condition at CSS = 1 showed a smaller intercept in the search function, which may reflect such search facilitation. Taken together, it is possible that, in the present study, attentional interference caused by relevant stimuli and search facilitation arising from the structure of the search display occurred simultaneously. Consequently, these opposing effects may have canceled each other out, preventing clear differences in search efficiency from emerging. Future studies should aim to more tightly control the structure of the search display to minimize facilitation effects, or employ experimental designs that allow the contributions of search facilitation and attentional capture to be disentangled. Such approaches would enable a clearer assessment of how relevant stimuli influence visual search performance.

Despite the null RT findings, recent advances in CIT research suggest that involuntary physiological markers—such as eye movements, pupil dilation, and neural oscillations—can provide more sensitive indicators of concealed information. For example, Millen et al. [14] demonstrated that the concealed recognition of familiar faces was reflected in multiple eye fixation metrics regardless of the participant’s honesty. Similarly, Rosenzweig and Bonneh [23] showed that even masked, passively viewed crime-relevant items modulated oculomotor inhibition in a CIT paradigm with high accuracy. These findings support the view that early ocular responses, which are relatively less susceptible to deliberate control, may be ideal candidates for detecting concealed knowledge. In future research, we propose incorporating such involuntary measures—especially oculomotor inhibition, fixation duration, and pupil dilation—into CIT paradigms using visual search.

## Conclusion

We investigated whether crime-related items would capture attention and impair visual search performance in three experiments employing a visual search paradigm. Although we replicated a previous study [12] using real objects as search items, we found no reliable evidence that related items influenced search efficiency. Further analysis indicated that the presence of related items in the search display did not affect the reaction times, even when the target appeared at their location. These findings suggest that in the absence of explicit task relevance, crime-related stimuli may not automatically guide attention. Future studies may benefit from incorporating physiological measures, such as eye tracking, to complement behavioral data and reveal more subtle effects.

## Data Availability

All raw data are available via [https://osf.io/y32ea/].

## Abbreviations

CIT: Concealed information test
RT: Reaction time
IOR: Inhibition of return
ANOVA: Analysis of variance
CSS: Cued set size
